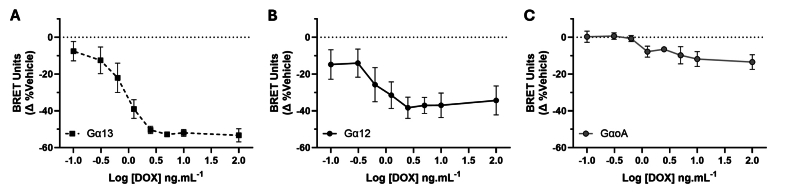# Biased constitutive signaling of the G protein-coupled receptor GPR35 suppresses gut barrier permeability

**DOI:** 10.1016/j.jbc.2024.108035

**Published:** 2024-11-29

**Authors:** Tezz Quon, Li-Chiung Lin, Amlan Ganguly, Brian D. Hudson, Andrew B. Tobin, Graeme Milligan

**Affiliations:** Centre for Translational Pharmacology, School of Molecular Biosciences, College of Medical, Veterinary and Life Sciences, University of Glasgow, Glasgow, Scotland, United Kingdom

**Keywords:** GPR35, constitutive activity, gut permeability, biased signaling

## Abstract

Agonist-independent, or constitutive, activity is an integral feature of G protein-coupled receptors, but its relevance in pathophysiological settings is generally poorly explored. GPR35 is a therapeutic target in inflammatory diseases of the lower gut. In colonic organoids from a human GPR35a-expressing transgenic mouse line, the GPR35 inverse agonist CID-2745687 increased barrier permeability substantially, indicating that constitutive receptor activity contributes to maintaining epithelial barrier integrity. High constitutive activity of GPR35 was also observed in both HT-29 and HEPG2 cells that express GPR35 endogenously. Mechanistic investigations in recombinant *in vitro* systems revealed that the constitutive activity of GPR35a was biased and not equivalent across signaling pathways. Hence, no constitutive interactions of the receptor with arrestin-adaptor proteins or activation of Gα_o_-containing G protein heterotrimers were detected while, even at low GPR35a expression levels, substantial constitutive activation of heterotrimers containing either Gα_12_ or Gα_13_ was observed. Similar biased constitutive activity was observed for the human GPR35b isoform. The extent of constitutive and agonist-mediated activity was dependent on receptor expression level. At high receptor levels, constitutive activation of Gα_12_ or Gα_13_ masked any agonist-induced effects while low expression levels with low constitutive activity allowed measurement of agonist-induced responses. These results highlight roles, selectivity, and the extent of constitutive activity of GPR35 in cells and tissues that express this receptor endogenously and highlight the contribution of its constitutive activity to maintaining the colonic epithelial barrier, potentially limiting the development of inflammatory bowel diseases.

G protein-coupled receptors (GPCRs) transition between multiple conformational forms in which active states are stabilized by the binding of agonist ligands and inactive states by binding of inverse agonists. The extent of basal (or constitutive) activity can vary markedly between individual GPCRs and can be important for physiological function and malfunction. For example, the light receptor rhodopsin displays very low constitutive activity, and mutations that increase this are associated with the development of retinitis pigmentosa (1). By contrast, the ghrelin receptor displays high levels of constitutive activity *in situ* (2) and suppression of its intrinsic activity has been suggested as a strategy to develop anti-obesity drugs (3).

GPR35 is a poorly understood member of the GPCR super-family that is attracting considerable interest as a therapeutic target in areas ranging from ulcerative colitis to digestive system cancers and neuropathic pain (4, 5, 6, 7, 8). Although officially an “orphan” receptor, meaning that its endogenous ligand(s) are uncertain or incompletely defined (9), GPR35 has a rich pharmacology of synthetic activators (10, 11, 12, 13, 14, 15, 16, 17). By contrast, antagonist/inverse agonist ligands are very limited in number and drug-like properties (7, 11) and available compounds with such activity only display affinity at the human but not rodent orthologues (18). This has resulted in many challenges in defining “on-target” effects of this receptor in rodent models of disease (4, 7) and how this receptor signals *via* different G protein-dependent pathways, as well as the extent of constitutive activity and if this may contribute to receptor function in various settings.

It is well established that agonists of GPR35 can promote activation of the G protein G_13_ (10, 19, 20, 21). Additionally, substantial literature has shown that, at least in certain cells and tissues, regulation of endpoints downstream of GPR35 activation is blocked by pretreatment of cells with Pertussis toxin (see 22 for review). This is diagnostic of the involvement of one or more of the “G_i/o_”-family of heterotrimeric G proteins (22). Indeed, in initial efforts to de-orphanize GPR35, ligand-induced binding of [^35^S]GTPγS was prevented by prior treatment with the Pertussis toxin of Chinese Hamster Ovary cells transfected to express the receptor (23). Additionally, in more recent studies, blockade of effects of GPR35 agonists by Pertussis toxin has been reported in systems ranging from mouse colonic epithelial cells (24) to cardiomyocytes (25). This apparent bifurcation in the signaling profile of GPR35 has not been addressed directly and has been largely overlooked by commentators. In addition, activation of GPR35 is associated with highly effective interactions with arrestin-adaptor proteins. This is reflected in many studies that explore and characterize ligand activation of GPR35 measuring such interactions (10, 11, 15, 16).

As noted earlier, there is considerable interest in assessing GPR35 as a therapeutic target in inflammatory bowel diseases because in Dextran Sodium Sulphate (DSS)-induced mouse models of colitis, activation of GPR35 is generally beneficial (17, 24, 26, 27), although such conclusions are not universal (28). However, the extent and contribution of constitutive activity of GPR35 to colon and lower gut barrier permeability has not been explored and neither has whether GPR35 promotes equally the constitutive activity of differing signaling cascades.

Here we demonstrate that the constitutive activity of GPR35 is important in maintaining colon epithelial barrier permeability and show a high level of constitutive activity of GPR35 in cell lines expressing the receptor endogenously. Such results support the potential benefits of GPR35 agonism, but not inverse agonism, in conditions with impaired mucosal barrier function. We then examine the relative capacity of the constitutive activity of GPR35 to activate G protein heterotrimers containing different α subunits and if this extends to other signaling pathways. We show that at low expression levels agonist-occupied GPR35 displays a marked preference for the G_12_/G_13_ pair of G proteins and that at higher receptor expression levels constitutive activity limits the capacity to observe agonist-mediated activation of G_12_. Moreover, although constitutive activation of the Pertussis toxin-sensitive G_o_-family G proteins was not detected across a wide range of receptor levels, at high receptor expression levels agonist-mediated activation of the isoforms of G_o_ is generated.

## Results

### GPR35 displays constitutive activity in endogenously expressing cell lines

**Figure 1:**
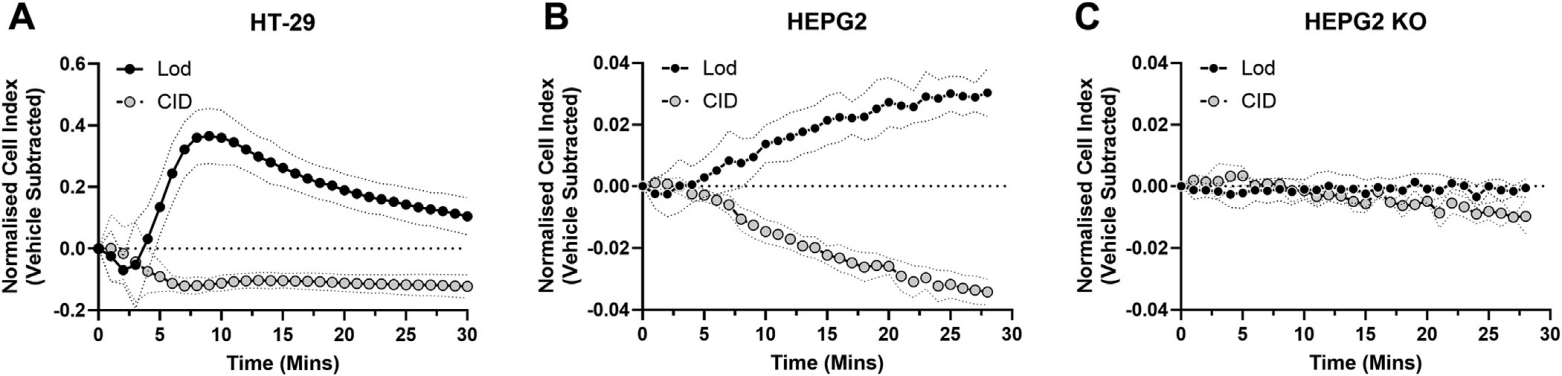
**hGPR35b displays constitutive activity in both HT-29 and HEPG2 cells.** Both HT-29 (*A*) and HEPG2 (*B*) cells express exclusively the hGPR35b isoform (31). Lodoxamide (Lod, 1 × 10^−6^ M) increased ‘cell index’ compared to vehicle in xCELLigence assays in both whilst CID-2745687 (CID, 1 × 10^−6^M) produced an opposite effect. *A*, *B*, effects of both lodoxamide and CID-2745687 were lacking in HEPG2 cells lacking GPR35b (31). *C*, data are the means of the means of three experiments, with SD as the error (*stippled lines*).

The human (h) colon adenoma cell line HT-29 has been used extensively in studies on the pharmacology and regulation of GPR35 (29, 30) and expresses exclusively the longer GPR35b isoform (31). In ‘label-free’ xCELLigence impedance assays that monitor alterations in electrical current across a cell monolayer then, compared to vehicle, lodoxamide (1 × 10^−6^M), a potent agonist of the hGPR35 isoforms (16), increased signal in a time-dependent manner (Fig. 1*A*). By contrast, the hGPR35 inverse agonist CID-2745687 (1 × 10^−6^M) (11) caused a decrease in signal that was maintained over time (Fig. 1*A*). This is consistent with hGPR35b displaying constitutive activity at endogenous expression levels that are suppressed by CID-2745687. Human hepatocyte-like HEPG2 cells also express exclusively the hGPR35b isoform (31). Although the pattern of responses in xCELLigence traces obtained from these cells (Fig. 1*B*) were distinct from those of HT-29 cells (compare Fig. 1, *A* and *B*), once more lodoxamide generated an increased signal whilst CID-2745687 produced a marked and time-dependent reduction (Fig. 1*B*). We have previously shown that such effects of lodoxamide in HEPG2 cells are prevented by the co-addition of CID-2745687 (31), indicating that they reflect the activation of GPR35. To further define that these effects of both lodoxamide and CID-2745687 were on-target and reflected alterations of basal GPR35-mediated signaling, we performed equivalent studies using HEPG2 cells that had been genetically modified to eliminate expression of the receptor (31). In these GPR35-knock-out HEPG2 cells, neither lodoxamide nor CID-2745687 produced an effect that was distinct from vehicle (Fig. 1*C*). In concert with previous studies in which we demonstrated that transient introduction of GPR35 into GPR35 knock-out HEPG2 cells restored the pattern and extent of xCELLigence signal to lodoxamide (31) this confirmed that the effect of CID-2745687 in wild-type HEPG2 cells was produced by suppression of the constitutive activity of hGPR35b.

### Physiological importance of GPR35 constitutive activity in regulating lower gut permeability

**Figure 2:**
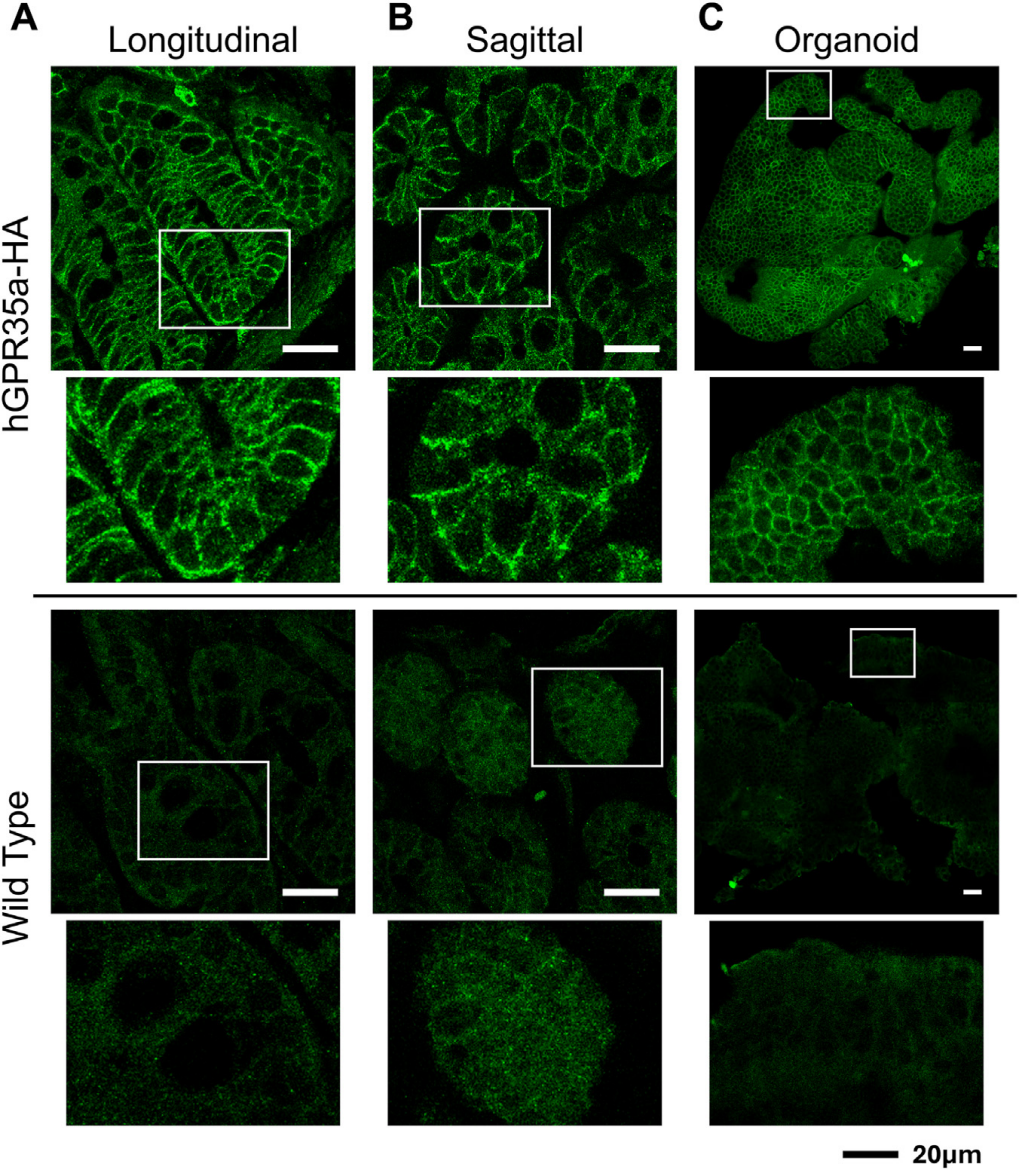
**hGPR35a is expressed in epithelial cells of colonic crypts and in colonoids derived from transgenic hGPR35a-HA mice.** Formalin fixed, paraffin-embedded longitudinal (*A*)- and sagittal (*B*)-sections of colons from hGPR35a-HA knock-in or wild type mice were stained with anti-HA antibody. Boxed areas in the *upper panels* are expanded in the *lower images*. Colon organoids in culture (*C*) generated from hGPR35a-HA knock-in or wild type mice maintained expression of hGPR35a-HA. Representative images are shown. Scale bars = 20 μm.

**Figure 3:**
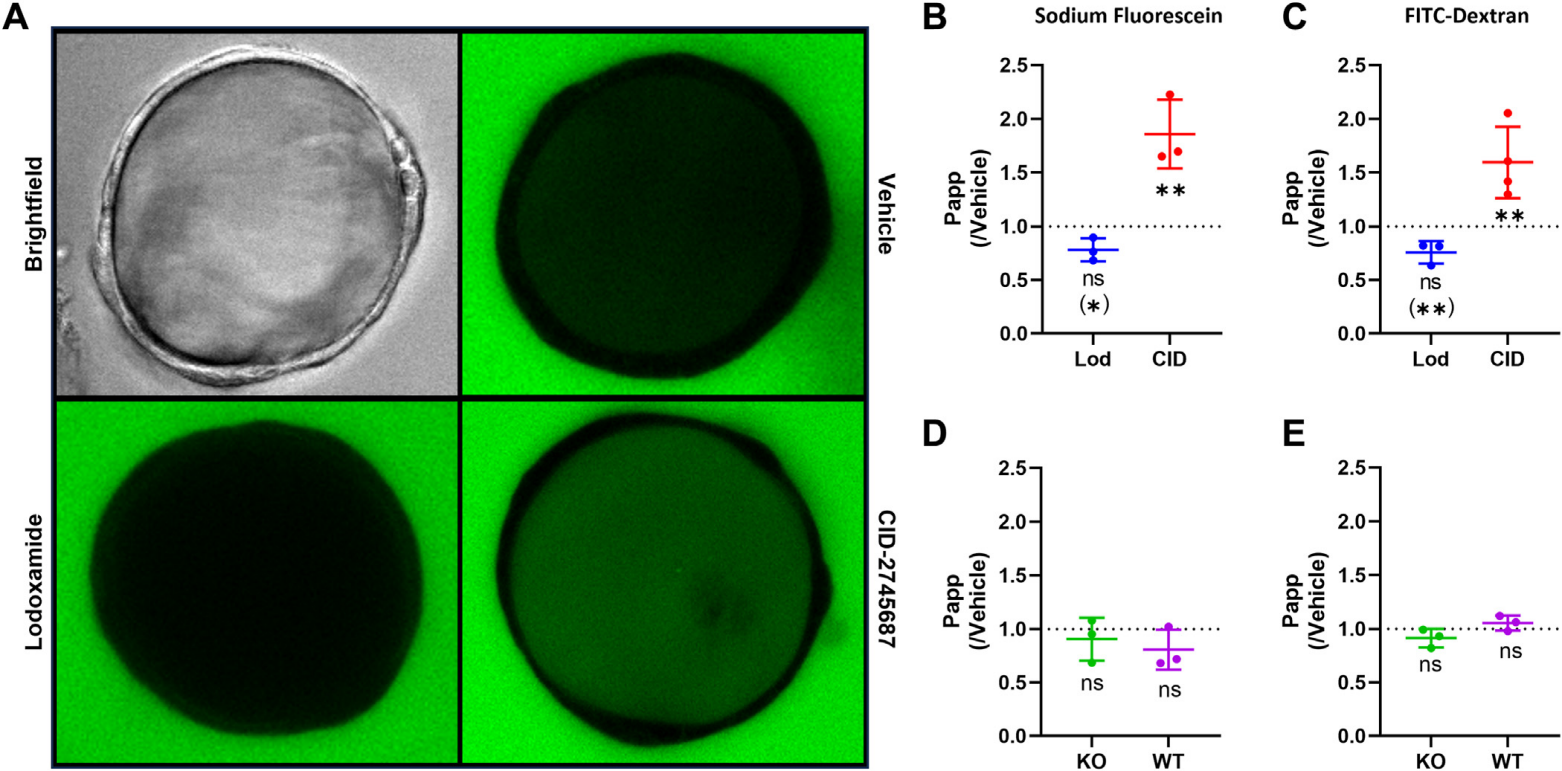
**Constitutive activity of hGPR35a regulates permeability in colonoids from hGPR35a-HA expressing mice.** hGPR35a-HA-derived colonoids (*A*) were treated for 16 h with either vehicle, lodoxamide (1 × 10^−6^M) or CID-2745687 (1 × 10^−5^M). These were then exposed to sodium fluorescein for 70 min and imaged using a confocal microscope. Representative images are shown. Permeability of sodium fluorescein (*B*) or FITC-dextran (*C*), measured as fluorescence intensity inside *versus* outside of colonoids, show lodoxamide produced a decrease in permeability compared to treatment with vehicle (Student’s *t* test (∗) *p* < 0.05, ANOVA ns). In contrast CID-2745687 significantly increased (∗∗, *p* < 0.01 Students *t* test & ANOVA) permeability. *D* and *E*, CID-2745687 lacked effect on barrier permeability in colonoids generated from either wild type (WT) or GPR35 knock-out (KO) mice. Data are means ± SD, n = 3, apart from (*C*), where lodoxamide n = 3 and CID-2745687, n = 4.

**Supplemental Figure 1:**
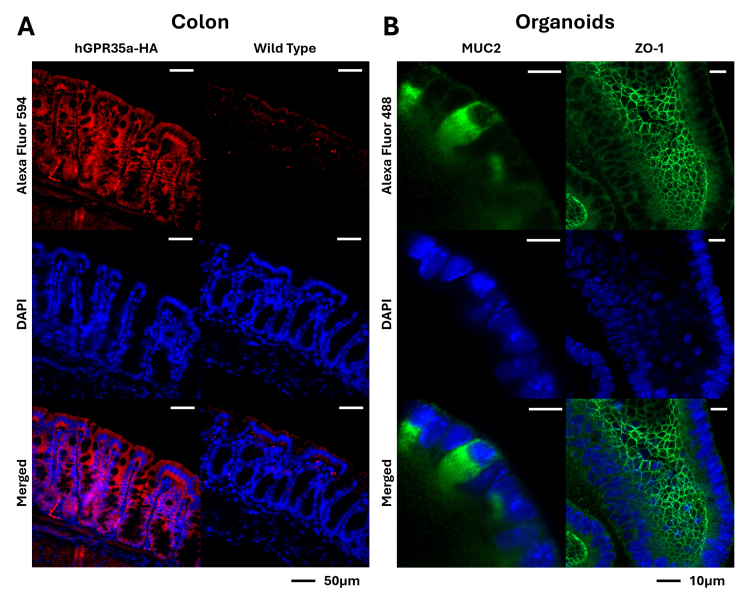


Although HT-29 and HEPG2 cells provide useful models to assess the function of endogenously expressed hGPR35 we wished to explore the constitutive- and ligand-regulated function of GPR35 in a physiologically important tissue. Mouse expresses a single form of GPR35 that corresponds to the shorter hGPR35a isoform. Because CID-2745687 lacks affinity at mouse GPR35 (18, 20) and lodoxamide has substantially lower potency at mouse GPR35 than at the human orthologue, we generated a transgenic knock-in mouse line in which mouse GPR35 was replaced by hGPR35a. In addition, we introduced an in-frame C-terminal haemagglutinin (HA)-epitope tag (31) to allow effective immuno-detection of the expression pattern of the receptor. Expression of the hGPR35a-HA construct in this line is driven by the mouse GPR35 promoter regions. Based on mRNA expression profiling hGPR35a is highly expressed in the colon of these mice (31). Immunostaining of colonic crypts of homozygous hGPR35a-HA animals with anti-HA showed intense staining corresponding to hGPR35a-HA, with particularly strong signal at cell-cell contact boundaries (Figs. 2, S1). Only background staining was observed in equivalent samples from wild type C57-Bl6 mice (Figs. 2, S1), confirming the specificity of the anti-HA staining for hGPR35a. To extend this and examine the activity of hGPR35a-HA *in situ* we generated colonoid cultures from both hGPR35a-HA knock-in and wild-type mice and again used anti-HA immunostaining to confirm maintained expression of the receptor (Fig. 2). Parallel detection of the tight junction protein ZO-1 confirmed the maintenance of appropriate cellular structure and organization in these colonoids (Fig. S1) whilst staining to detect mucin 2 (MUC2) which forms an insoluble mucous barrier that protects the gut lumen confirmed the maintained presence of this key polypeptide (Fig. S1). We then assessed permeability of these colonoids (Fig. 3), initially to sodium fluorescein. Basal permeability was modest but reduced by exposure to lodoxamide (1 × 10^−6^M, 16h) (*p* < 0.05) (Fig. 3, *A* and *B*). Notably, equivalent exposure to CID-2745687 (1 × 10^−6^M, 16h) resulted in significantly increased (*p* < 0.01) permeation of sodium fluorescein (Fig. 3, *A* and *B*). As an extension, equivalent studies were performed using FITC-dextran, and these produced similar results (Fig. 3*C*) with CID-2745687 again significantly (*p* < 0.01) increasing permeability. To confirm that this effect of CID-2745687 reflected hGPR35a-HA-mediated constitutive activity we generated equivalent colonoid cultures from both GPR35 knock-out mice and from wild-type mice. In both these sets of colonoids, CID-2745687 did not enhance the permeability of either sodium fluorescein (Fig. 3*D*) or FITC-dextran (Fig. 3*E*). The effect of CID-2745687 in colonoids generated from hGPR35a-HA expressing mice indicates that, at endogenous expression levels, the constitutive activity of the receptor limits colonic permeability. The reduction of permeability in colonoids from hGPR35a-HA mice by treatment with lodoxamide suggests that activators of GPR35 might be useful in the treatment of lower gut inflammatory conditions, such as ulcerative colitis, in which mucosal barrier function is compromised. By contrast, GPR35 inverse agonists would be contra-indicated. In wild-type mice, the lack of effect of CID-2745687 reflects that this ligand lacks affinity for mouse GPR35 (18, 20) and studies on agonist effects require ligands, which, unlike lodoxamide, show high potency at the mouse orthologue.

### Defining mechanisms of hGPR35 constitutive activity

**Figure 4:**
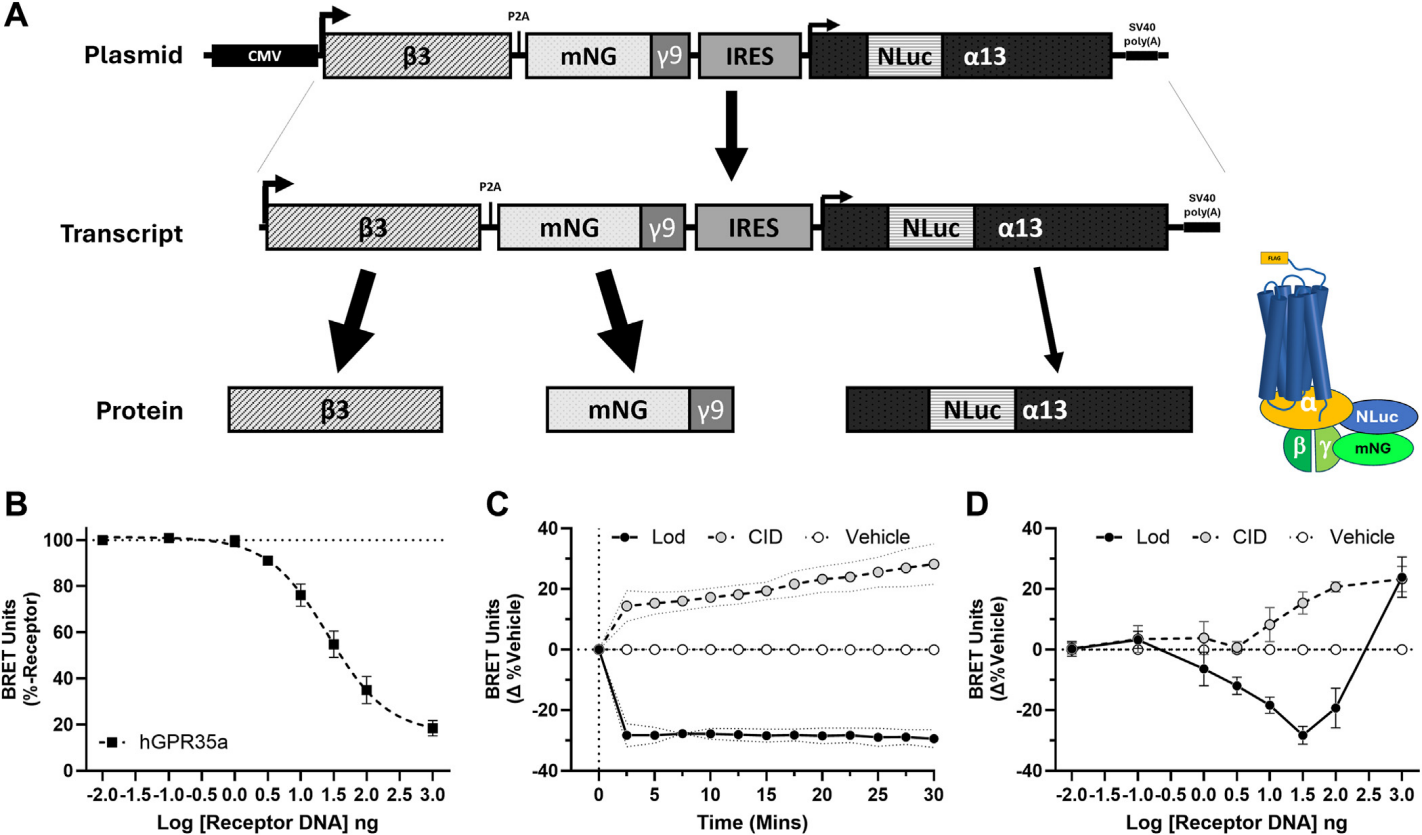
**hGPR35a is able to promote both constitutive and agonist-induced activation of Gα**_**13**_**.** A modified TRUPATH (52)-like construct able to express a G protein heterotrimer consisting of Gβ3, mNeonGreen (mNG)-γ9, and Gα_13_ with an internally located Nanoluciferase (NLuc) subunits was generated. The Gβ3 and mNG-γ9 sequences were separated by the self-cleavage sensitive P2A peptide and were upstream of an IRES sequence which was followed by the NLuc-tagged Gα_13_. The cartoon (*A*) illustrates the components of the BRET sensor anticipated to be generated post-transfection and expression. *B*, HEK293 cells were transiently transfected with the Gα_13_-containing sensor (5 μg) and the indicated amounts of FLAG-hGPR35a. BRET, reflecting the extent of association of the G protein heterotrimer was then measured. *C*, HEK293 cells co-transfected with 31.6 ng FLAG-hGPR35a plasmid and the Gα_13_-containing sensor were exposed to vehicle (*open circles*), the GPR35 agonist lodoxamide (1 × 10^−6^M) (*dark circles*) or the hGPR35 inverse agonist CID-2745687 (1 × 10^−5^M) (*light circles*) and alteration in BRET signal (relative to vehicle treated) measured over time following addition of a luciferase substrate. *D*, varying amounts of the FLAG-hGPR35a plasmid were transfected into HEK293 cells along with a constant amount of the TRUPATH sensor plasmid. Effects of CID-2745687 or the hGPR35 agonist lodoxamide (each at 1 × 10^−5^M) on BRET signal were measured after 5 min and compared to treatment with vehicle. Data are means ± SD, n = 3.

**Supplemental Figure 2:**
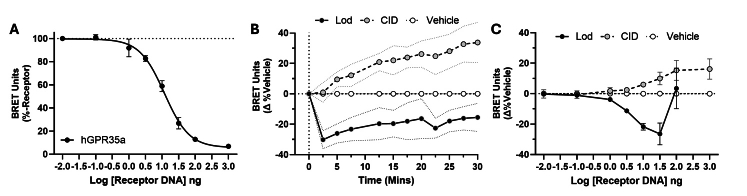


The data above show the presence and effects of constitutive activity of hGPR35 isoforms in both cells and tissue expressing the receptor endogenously. However, it is impractical to dissect the key cellular signaling mechanisms involved without employing heterologous expression systems that allow receptor expression levels to be modulated and to assess if different G proteins and signaling adaptor proteins are activated to similar extents by the constitutive activity of hGPR35. It is well established that hGPR35 can activate the G protein G_13_ (18, 20). As such, we initially transiently co-transfected into HEK293 cells a fixed amount of an Internal Ribosome Entry Site (IRES)-based plasmid that allows co-expression of each of a Nanoluciferase (NLuc)-tagged form of Gα_13_, Gβ3, and a mNeonGreen (mNG)-tagged form of Gγ9 (Gα_13_/Gβ3/Gγ9 heterotrimer) (Fig. 4*A*) and varying amounts of cDNA encoding a N-terminally FLAG-tagged form of hGPR35a. Subsequently, we measured Bioluminescence Resonance Energy Transfer (BRET) between the modified forms of Gα_13_ and Gγ9 because this provides information on the association state of the G protein complex. BRET declined markedly with increasing levels of the receptor (Fig. 4*B*). This required expression of the receptor and confirmed that hGPR35a can promote activation of Gα_13_ and its dissociation from the β3/γ9 complex in the absence of a receptor agonist. Based on the apparent IC_50_ for receptor cDNA (Fig. 4*B*) we then co-transfected HEK293 cells with the Gα_13_/Gβ3/Gγ9 heterotrimer along with 31.6 ng receptor plasmid DNA with the anticipation that in addition to a level of constitutive activity this might allow detection of effects of both GPR35 agonists and inverse agonists. Compared to vehicle-treated cells, the addition of lodoxamide (1 × 10^−6^ M) further reduced the BRET signal, and this reduction was maintained over time (Fig. 4*C*). This is consistent with previous reports that agonist occupancy of hGPR35a promotes interaction with Gα_13_ (20, 21) and subsequent enhanced dissociation of the G protein α subunit and β/γ complex. Notably, in these conditions, the addition of CID-2745687 (1 × 10^−5^M) instead increased BRET (Fig. 4*C*) in a manner that was sustained and, indeed, increased somewhat over time. This is also consistent with CID-2745687 suppressing the ligand-independent constitutive activity of the receptor to allow time-dependent if apparently rather slow, re-association of the G protein heterotrimer. To explore this effect of CID-2745687 more fully we again co-transfected the Gα_13_-containing IRES plasmid with differing amounts of FLAG-hGPR35a plasmid DNA. At a fixed 5 min time point the BRET signal induced by CID-2745687 increased with receptor plasmid amount (Fig. 4*D*). Interestingly, although at low hGPR35a plasmid levels addition of lodoxamide markedly reduced BRET signal, this was less extensive at higher receptor plasmid DNA amounts and, indeed, was absent at the highest amounts of receptor plasmid utilized (Fig. 4*D*). Equivalent behavior was also observed when the Gα_13_/Gβ3/Gγ9 heterotrimer was replaced with a corresponding G protein heterotrimer containing Gα_12_ (Fig. S2). This group of observations indicates modest constitutive activation and marked agonist-dependent activation of both Gα_12_ and Gα_13_ G proteins at low receptor levels but substantial constitutive activation, which thus limits the potential for additional agonist-dependent effects, at higher receptor levels.

### Constitutive activity of hGPR35 promotes activation of only a subset of G proteins

**Figure 5:**
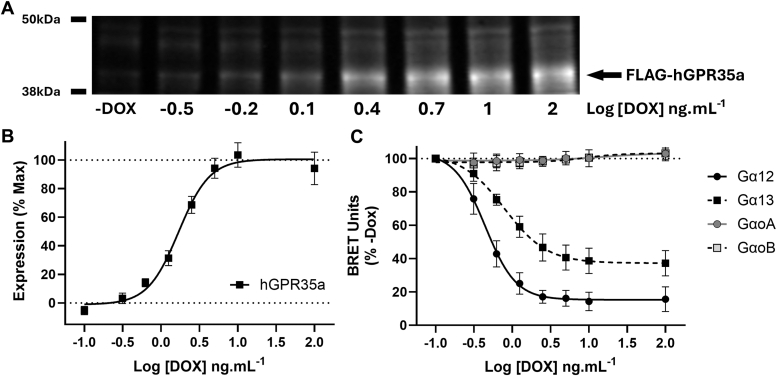
**Varying hGPR35a levels highlight receptor-induced constitutive activation of both Gα**_**13**_**and Gα**_**12**_**but not Gα**_**o**_**isoforms.** A Flp-In T-REx 293 cell line stably harboring FLAG-hGPR35a was established. Following treatment of cells for 16 h with varying concentrations of doxycycline, expression of the receptor was assessed by immunoblotting cell lysates with anti-FLAG (*A*). FLAG-hGPR35a is highlighted by the arrow. A representative example is shown. In-Cell Western blots to detect the N-terminal (and hence extracellular) FLAG-tag were quantified (*B*). Data are means ± SD, n = 3. Following transient expression of Gα_13_/Gβ3/Gγ9 (*dark squares*), Gα_12_/Gβ3/Gγ9 (*dark circles*), Gα_oA_/Gβ3/Gγ9 (*light circles*) or Gα_oB_/Gβ3/Gγ9 (*light squares*) into cells treated with the indicated concentrations of doxycycline (DOX), BRET was measured 10 min after addition of the NLuc substrate (*C*). Ligand-independent constitutive activity was observed for Gα_12_ and Gα_13_, but not for either Gα_oA_ or Gα_oB_. Data are means ± SD n = 6 and presented as NLuc corrected BRET units normalized to no DOX-induced FLAG-hGPR35a expression.

To overcome potential variable expression in individual transient transfections and to explore in detail whether the constitutive activity of hGPR35 would activate all G protein subtypes equally we established a Flp-In T-REx 293 cell line able to express varying amounts of FLAG-hGPR35a with exposure to differing concentrations of doxycycline (32). The variation in receptor expression was measured both by immunoblotting lysates of these cells with an anti-FLAG antibody (Fig. 5*A*) and by ‘in-cell’ Western blotting to quantify receptor levels (Fig. 5*B*). These both showed substantial alteration in hGPR35a expression levels when cells had been exposed to between 0.3 and 3 ng mL^−1^ of doxycycline over a 16 h period. After treatment with higher doxycycline concentrations further increase in the amount of hGPR35a was minimal (Fig. 5, *A* and *B*). Now, additional transient transfection of these cells to express the Gα_13_/Gβ3/Gγ9 heterotrimer resulted in marked constitutive activation of the G protein with doxycycline concentrations above 0.3 ng mL^−1^ (Fig. 5*C*). This plateaued at doxycycline concentrations above 3 ng mL^−1^ (Fig. 5*C*). The extent of constitutive G protein activation in this system was even more marked when the Gα_13_/Gβ3/Gγ9 heterotrimer was replaced with the corresponding Gα_12_/Gβ3/Gγ9 heterotrimer (Fig. 5*C*). Such effects were not observed, however, for all G protein α subunits. For example, co-expression of either a Gα_oA_/Gβ3/Gγ9 complex or a Gα_oB_/Gβ3/Gγ9 complex did not result in their constitutive activation across the full range of levels of hGPR35a that could be induced (Fig. 5*C*).

### hGPR35a constitutive activity masks agonist activation of G_12_ and G_13_ proteins

**Figure 6:**
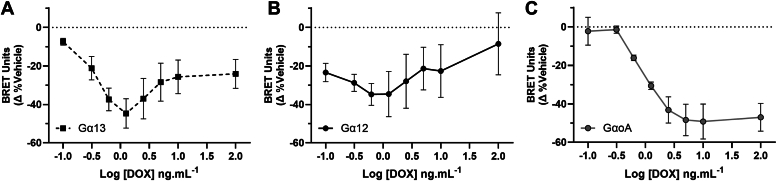
**Agonist-induced activation of Gα**_**oA**_**isoforms is only observed at higher hGPR35a levels than required to activate Gα**_**12**_**or Gα**_**13**_**.** Lodoxamide (1 × 10^−6^M) promotes activation and dissociation of both Gα_13_/Gβ3/Gγ9 (*A*) and Gα_12_/Gβ3/Gγ9 (*B*) at low DOX (and hence receptor) levels and this is limited or reverses at higher DOX concentrations. Activation of Gα_oA_/Gβ3/Gγ9 (*C*) required higher levels of receptor and was maintained with increasing receptor amount. NLuc corrected BRET units normalized as % change from the vehicle. Data are means ± SD, Gα_12_ and Gα_13_, n = 4, Gα_oA_, n = 3.

We next examined the effects of varying hGPR35a expression levels on agonist-mediated G protein activation. Here lodoxamide induced Gα_13_/Gβ3/Gγ9 activation/dissociation at low hGPR35a levels (Fig. 6*A*). As hGPR35a expression increased with increasing concentrations of doxycycline then agonist effects on Gα_13_/Gβ3/Gγ9 activation initially similarly increased (Fig. 6). However, this effect reached an inflection point at 1 ng.mL^−1^ doxycycline and at higher levels agonist effects were reduced. This inflection point corresponded to hGPR35a expression levels sufficiently high to result in measurable constitutive activity (Fig. 5*C*). Hence, at higher receptor expression levels constitutive activity of hGPR35a limits detection of additional agonist activation of this heterotrimeric G protein complex (Fig. 6*A*). These features were even more pronounced for Gα_12_/Gβ3/Gγ9 where no lodoxamide-mediated activation of this G protein was detected at high receptor levels (Fig. 6*B*). By contrast, although no lodoxamide-induced dissociation/activation of Gα_oA_/Gβ3/Gγ9 could be observed at low FLAG-hGPR35a levels (Fig. 6*C*) this was readily detectable at higher FLAG-hGPR35a levels (Fig. 6*C*). Moreover, because of the lack of constitutive activation of the Gα_o_-containing heterotrimers (Fig. 5*C*), the extent of the effect of lodoxamide at these G proteins was maintained at the highest levels of receptor expression achieved (Fig. 6*C*).

**Figure 7:**
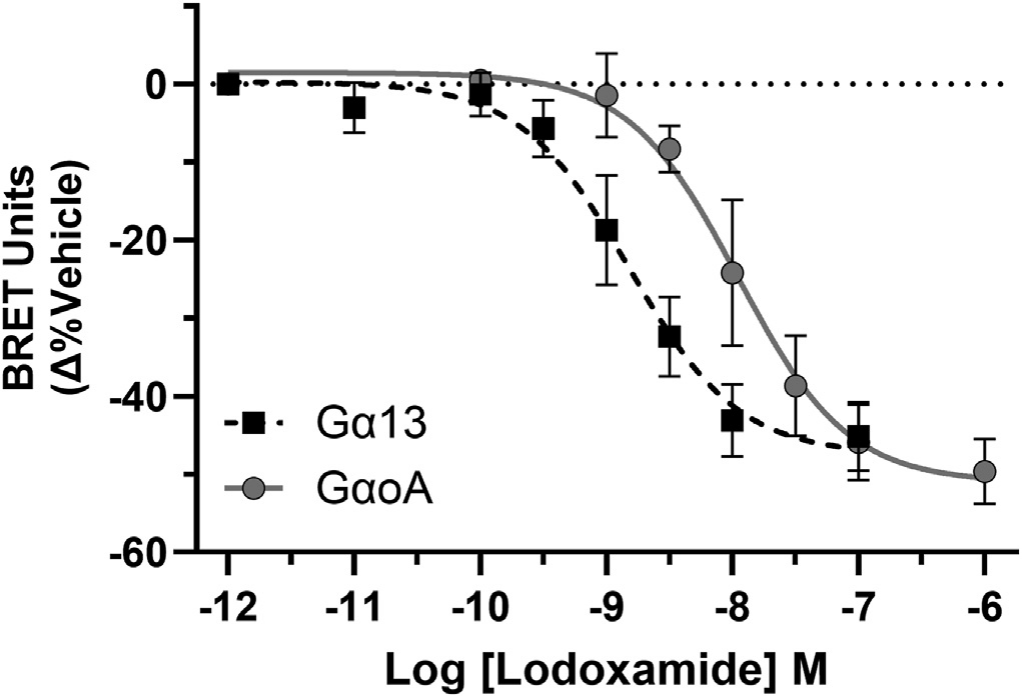
**The potency of lodoxamide to activate Gα**_**13**_**is greater than for the Gα**_**o**_**isoforms.** The Flp-In T-REx 293 cell line able to inducibly express FLAG-hGPR35a was treated with either 1.25 ng (Gα_13_) or 10 ng (Gα_oA_) doxycycline.mL^−1^ for 16h and following transient expression of Gα_13_/Gβ3/Gγ9 (*dark squares*) or Gα_oA_/Gβ3/Gγ9 (*light circles*) heterotrimeric complexes assessed for the ability of varying concentrations of lodoxamide to promote the activation and dissociation of these G proteins. Lodoxamide was significantly more potent (*p* < 0.001) in activating Gα_13_. Data are means ± SD, Gα_13_, n = 5, Gα_oA_, n = 3.

At expression levels of hGPR35a titrated to optimize the detection of agonist-mediated effects lodoxamide was almost 10-fold more potent (*p* < 0.0001) in activating the Gα_13_-containing complex (pEC_50_ = 8.83 ± 0.08) than the Gα_o_ complex (pEC_50_ = 7.95 ± 0.11) (Fig. 7). This indicates that in addition to causing selective constitutive activation of Gα_12_ and Gα_13_ lodoxamide-occupied hGPR35a interacts selectively with Gα_13_ compared to Gα_oA_. Previous studies have indicated that GPR35 isoforms are unable to activate G_q_-family G proteins (20, 21). In agreement with this, neither constitutive nor lodoxamide-mediated activation of G_q_/Gβ3/Gγ9 or G_11_/Gβ3/Gγ9 complexes was observed in equivalent experiments using hGPR35a (**not shown**). These studies imply that at endogenous levels of expression of GPR35 G protein-mediated effects *via* G_12_/G_13_ will likely be favored.

### hGPR35a displays constitutive signaling selection-bias

**Figure 8:**
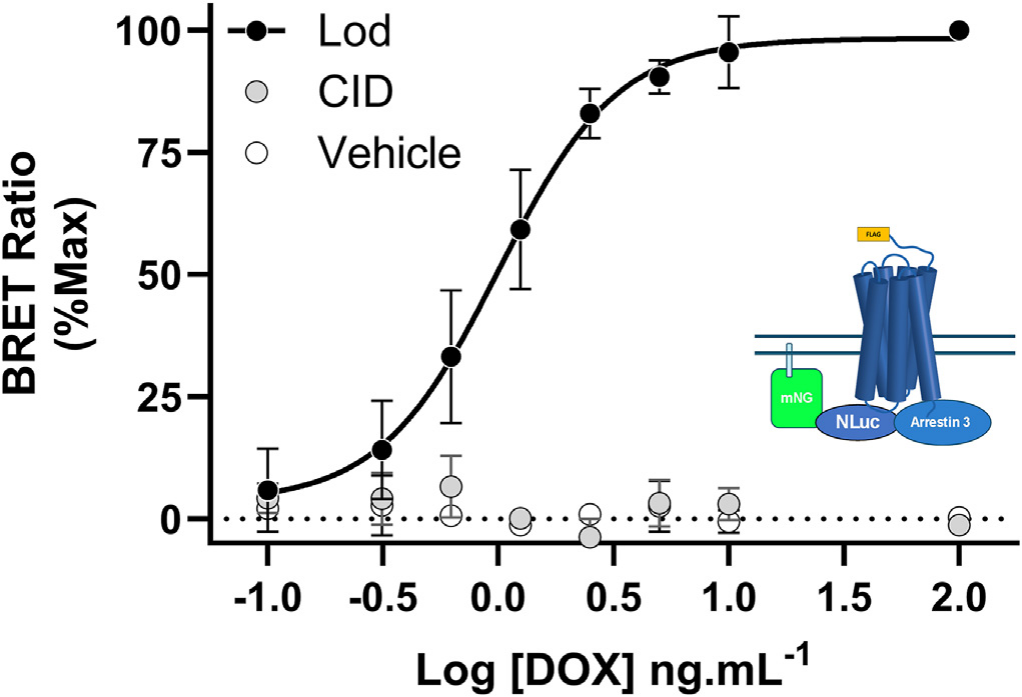
**Constitutive activity of GPR35a is not detected in a β-arrestin-2 recruitment assay.** The ability of lodoxamide (1 × 10^−6^M) (*dark circles*) or CID-2745687 (1 × 10^−5^M) (*light circles*) to modulate interactions between FLAG-hGPR35a and N-Luc β-arrestin-2 (arrestin 3) was assessed. The cartoon illustrates the basis of the bystander assay with nNeonGreen targeted to the cell surface membrane. No significant constitutive interaction/proximity of FLAG-hGPR35a with β-arrestin-2 was observed at any FLAG-hGPR35a expression level achieved, although lodoxamide robustly promoted such interactions. CID-2745687 was without effect. Data are means ± SD, n = 3.

In addition to selection between G protein classes, receptor-ligand “bias” in GPCR signaling is often described in terms of differential interactions between G proteins and arrestin-adapter proteins. β-arrestin interaction/proximity assays have been widely used to explore and define the pharmacology of GPR35 ligands (10, 11, 16, 19, 21, 33, 34). To take advantage of the doxycycline-mediated control of hGPR35a levels in the Flp-In T-REx cell line we assessed receptor-β-arrestin-2 proximity using receptor ‘bystander’ (35) assays. Here, both a plasma membrane-targeted form of mNeonGreen and NLuc-tagged β-arrestin-2 were transiently co-expressed in these cells at varying receptor expression levels. Whilst an extremely robust increase in BRET signal was generated upon addition of lodoxamide as receptor expression levels were increased (Fig. 8), no detectable agonist-independent signal was observed at any receptor expression level achieved and addition of CID-2745687 was without effect (Fig. 8). Hence, hGPR35a constitutive activity displays marked signaling selection-bias and the receptor only engages with arrestins in an agonist-dependent manner. This is consistent with previous reports that basal, agonist-independent levels of both total receptor phosphorylation and phosphorylation of key Ser/Thr residues in the C-terminal tail of hGPR35a are undetectable (33, 34).

### Comparison of hGPR35a and hGPR35b isoforms

**Supplemental Figure 3:**
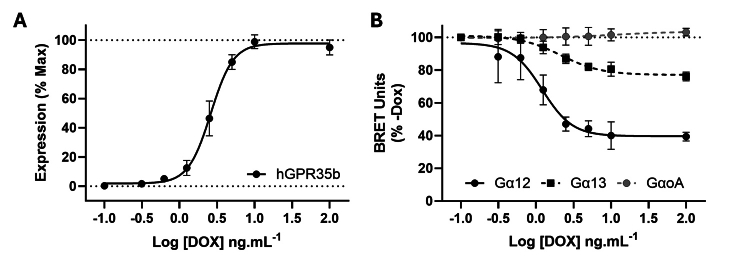


Compared to hGPR35a, hGPR35b contains a 31 amino acid N-terminal extension (13, 36). When induced to varying levels in equivalent stably transfected Flp-In T-REx 293 cells (Fig. S3*A*) hGPR35b also showed constitutive activation of both Gα_13_/Gβ3/Gγ9 (Fig. S3*B*) and, particularly, Gα_12_/Gβ3/Gγ9 (Fig. S3*B*) but not Gα_oA_/Gβ3/Gγ9 (Fig. S3*B*). In this way, each human GPR35 isoform behaved similarly. In contrast to hGPR35a, however, the “masking” of the agonist effects of lodoxamide on Gα_12_/Gβ3/Gγ9 and Gα_13_/Gβ3/Gγ9 by constitutive activity associated with high levels of receptor expression seen for hGPR35a (Fig. 6) was not evident for hGPR35b (Fig. S4). Rather the effects of agonist on these heterotrimeric G proteins plateaued at high receptor expression levels (Fig. S4). Interestingly, coupling of hGPR35b to Gα_oA_/Gβ3/Gγ9 appeared to be poor because in addition to a lack of constitutive activation of this G protein complex by hGPR35b (Fig. S3*B*) the extent of lodoxamide-mediated activation was substantially lower than for hGPR35a (Fig. S4).

## Discussion

GPCRs move through various conformational states to transition from inactive to active. The basal balance between such states can vary markedly between receptors and be altered by mutations and other variations in receptor sequence or post-translational modifications (1). Real, or apparent, constitutive or ligand-independent activity has been recorded for many GPCRs. However, particularly for orphan receptors, where the endogenous ligand(s) is either unknown or poorly defined, and in native settings, it is frequently challenging to assess if such measures are compromised because an unidentified activator may be present. Recent structural insights, for example on GPR3, where electron density potentially corresponding to a “lipid-like” molecule was noted, led to the suggestion that such a ubiquitously present ligand might be responsible for measured constitutive activity (37). This is conceptually similar to questions posed many years ago about the potential for undetected fatty acids to generate apparent constitutive activity at Free Fatty Acid Receptor 1 (38, 39).

A range of potential endogenous activators of GPR35 have been proposed. These include 5-HIAA (40), CXCL17 (41), and lysophosphatidic acid (42, 43) but, to date, the only endogenously produced ligand shown by multiple groups to activate GPR35, and in settings from transfected cells to tissues, is the tryptophan metabolite kynurenic acid (see Refs. (2, 7, 44) for review). Moreover, although the constitutive activity of both the short (GPR35a) and long (GPR35b) isoforms of hGPR35 has been explored previously in terms of each of interactions with G proteins, downstream signaling components and, indeed, regulation of longer-term reporter gene constructs (21) this has generally focused on simple heterologous transfections into lines such as HEK293 cells. We have now explored the extent of constitutive activity of GPR35 expressed endogenously in well-studied human-derived cell lines and in colonic tissue taken from a transgenic knock-in mouse line in which we replaced the sequence of mouse GPR35 with that corresponding to the equivalent human isoform, GPR35a. The focus on the human-derived cell lines reflects that GPR35 has been well studied in both HT-29 and HEPG2 cells (31, 45) and because the ligand CID-2745687 (11), while a high-affinity inverse agonist at hGPR35, has no significant affinity at either rat or mouse GPR35 (18, 20). Tissue from the human GPR35a-expressing mouse line was of particular interest because in these animals receptor expression is driven from the mouse *Gpr35* promoter sequences and this results in similar levels of mRNA encoding human GPR35a as for mouse GPR35 in wild-type animals (31).

In simple co-transfection studies, agonist-independent activation of G protein heterotrimers containing Gα_12_ and Gα_13_, that was ablated in the presence of CID-2745687 was extensive. However, whilst the extent of the effect of CID-2745687 increased with levels of co-transfected hGPR35a, at higher receptor levels further G protein activation and dissociation in response to the agonist lodoxamide was instead reduced. We interpret this to reflect that with greater constitutive G protein activation there is less remaining associated G protein α/βγ complex that can interact with the agonist-occupied receptor to become activated. While transient co-transfection made this difficult to fully define and quantify, the same pattern was observed using Flp-In T-REx 293 cell lines in which receptor expression is controlled by exposure to varying concentrations of doxycycline (32). Over the doxycycline concentration range that resulted in variation in receptor expression constitutive activation of both Gα_12_ and Gα_13,_ but not isoforms of Gα_o_, was observed, and this was true for both hGPR35a and hGPR35b as well as both rat and mouse GPR35 (**data not shown**). For hGPR35a notably, although no constitutive activation of Gα_o_ was observed at any receptor level achieved, the G protein containing this α-subunit was activated in an agonist-dependent manner, but only at higher levels of receptor activation than required to do so for Gα_13_. We explored such G protein α subunit preference using only a single (Gβ3/Gγ9) β/γ combination and this may have contributed to the observed outcomes. This is an area that deserves further attention but trying to define which β/γ combinations exist in different cell types is challenging, and to assess if this genuinely might alter the functional responses of receptors remains a major, and possibly impractical, undertaking. Interestingly, the extent of lodoxamide activation of Gα_o_ was less pronounced in Flp-In T-REx 293 cells induced to express hGPR35b. While Schihada *et al.* (21), did not use lodoxamide as an agonist, they did employ each of pamoic acid, kynurenic acid, and zaprinast but did not observe ligand-induced activation of Gα_oA_ or other Pertussis toxin-sensitive G proteins. This is perhaps surprising as there is substantial literature on the Pertussis toxin-sensitive effects of GPR35 agonists in a range of cells and tissues (see Ref. (46) for review).

Such transfected cell systems offer a means to explore both agonist-independent and agonist-dependent effects of isoforms and species orthologues of GPR35 but are likely to be performed at supra-physiological receptor levels. Moreover, they offer no insight into the extent and potential relevance of the constitutive activity of GPR35 in cells and tissues natively expressing the receptor. Prior to examining animal tissue, we explored the constitutive *versus* agonist-mediated activity of GPR35 in both human HT-29 adenocarcinoma and hepatocyte-like HEPG2 cells. Both are known to express exclusively the GPR35b isoform (31). We employed so-called “label-free” xCELLigence analysis because changes in receptor-mediated outputs can be measured in a sustained manner over time and are believed to integrate cell signals to produce the output. In each case, we employed ligand concentrations shown previously to be close to maximally effective (31). For HT-29 cells the effect of the inverse agonist CID-2745687 was in the opposite direction to that produced following the addition of the agonist lodoxamide. This is consistent with CID-2745687 suppressing constitutive activity of endogenously expressed GPR35b. Furthermore, whilst similar alterations in the direction of the signal were induced by lodoxamide and CID-2745687 in parental HEPG2 cells, neither ligand produced a response significantly different from the application of vehicle in a clone of HEPG2 cells genome-edited to lack expression of GPR35 that we have previously characterized in detail (31). This indicates that the effects of both lodoxamide and CID-2745687 are produced *via* occupancy of GPR35b and that CID-2745687 in wild-type HEPG2 cells is acting to suppress receptor constitutive activity.

GPR35 is attracting interest in various therapeutic areas (7), with suggestions that GPR35 agonists may be beneficial in the repair of mucosal barrier function that is damaged in inflammatory conditions of the lower gut (24). This reflects that one tissue with substantial levels of GPR35 is the colon (47, 48, 49, 50), that in the majority of studies elimination of GPR35 from mice increases severity of Dextran-Sodium Sulphate-induced colitis and that drugs that are used in this area, including amino-salicylates, target GPR35 to generate at least part of their effect (17, 27). To define the potential contribution of the constitutive activity of GPR35a to colon permeability we generated colonic organoids from a hGPR35a-expressing transgenic mouse line (31). As GPR35a expressed by these mice includes an engineered HA-epitope tag sequence we were able to define and highlight the cellular expression pattern. Here, whilst some of the detected receptor is at intracellular locations, much of it is present at surface cell-cell contacts. Importantly, expression of GPR35a-HA was maintained in the colonic organoids derived from the mice. By measuring permeability to either FITC-dextran or sodium fluorescein we observed that although the agonist lodoxamide reduced basal permeability, CID-2745687 increased this significantly. Once more, the effect of CID-2745687 was clearly mediated *via* GPR35a because the inverse agonist was without effect on permeability in equivalent colonic organoids produced from GPR35 knock-out mice. Potentially of equal importance, because CID-2745687 has no affinity for mouse GPR35, CID-2745687 also did not increase the permeability of colonic organoids isolated from wild-type animals.

A challenge in unambiguously defining that the effect of CID-2745687 in colonic organoids reflects suppression of the constitutive activity of GPR35 is that GPR35 remains an “orphan” receptor (7). Although various proposed endogenous activators of this receptor, such as CXCL17 (47), have failed to be confirmed and others, such as 5-HIAA (40), remain to be fully assessed (7), a caveat to some of our conclusions is that CID-2745687 may be acting to block the effect of an undetected and undefined endogenous ligand. Such concepts are hard to rule out but since effects of CID-2745687 were observed across transiently and stably transfected cell lines, in two distinct cell lines that express the receptor endogenously and in colonoids isolated from mice, this suggests that high-level receptor constitutive activity is the more likely explanation.

These studies provide a comprehensive analysis of the extent of constitutive-compared to agonist-regulated GPR35 activity across the two human isoforms. Importantly, as well as exploring how these affect activation of different G proteins at varying receptor expression levels, we also demonstrate the extent of constitutive activity of the long GPR35b isoform expressed endogenously in a pair of cell lines that are widely employed and well-studied as a cancer cell model (HT-29) and to explore compound toxicity in drug development (HEPG2) and to provide a model of the potential use of GPR35 agonists to limit fatty liver disease (31, 45, 51). Finally, we translate understanding and approaches to indicate that constitutive activity of GPR35a limits mucosal barrier permeability, and that blockade of this activity would be anticipated to worsen symptoms associated with inflammatory bowel diseases.

## Experimental procedures

### Materials

Reagents were obtained from the following suppliers: L-WRN Cells (ATCC, CRL-3276), Linear polyethylenimine (PEI), 25,000 MW (PolySciences, 23,966), doxycycline (DOX) (Sigma, D5207), Mouse EGF recombinant protein (EGF) (ThermoFisher, PMG8041), primocin (Invivogen, ant-pm-1), ROCK-Inhbitor Y-27632 (HelloBio, HB2297), matrigel (Corning, 356,237), coelenterazine-h (NanoLight, 301), FITC-Dextran 4000 Mw (Sigma, FD4), sodium fluorescein (Sigma, 46,960–100G-F), VECTASHIELD Antifade mounting medium with DAPI (Vectorlabs, H-1200–10), Protease Inhibitor Cocktail (Roche, 5892970001), Pierce BCA Protein Assay Kit (ThermoFisher, 23,225), Laemmli buffer (Sigma, 53,401-1VL), NuPAGE Bis-Tris Mini Protein Gels, 4 to 12%, 1.0 to 1.5 mm (ThermoFisher, NP0321BOX), Nitrocellulose membranes (ThermoFisher, 88,018), Cell-Tag 700 (LI-COR Biosciences, 926–4109). The GPR35 ligands lodoxamide, CID-2745687, and zaprinast were obtained from Tocris Bioscience.

### Plasmid construction

The *Renilla* luciferase (RLuc) sequence in the human Gα subunits from the published TRUPATH constructs (52), (Addgene #1000000163) was replaced with Nanoluciferase (NLuc) and cloned between the XbaI and NotI sites following the Internal Ribosome Entry Sequence (IRES) sequence in the pIRES vector. Green Fluorescent Protein 2 (GFP2) was replaced with mNeonGreen in the GFP2-γ9 (human gamma 9, GNGT2) TRUPATH construct and cloned in-frame with human β-3, separated by the self-cleavage peptide P2A (GSGATNFSLLKQAGDVEENPGPGSG), upstream of the IRES sequence between the NheI and MluI sites. These final sensor constructs are reminiscent of the G-CASE sensors (53) in that they are tri-cistronic sensors, though these use the same insert sites and linkers as the Trupath sensors. Receptor constructs were generated by cloning the coding sequences of hGPR35a or hGPR35b, each with an in-frame N-terminal FLAG epitope tag (DYKDDDDK), into the pcDNA5.1 FRT/TO vector.

The Gα_13_-NLuc construct in pcDNA3.1 was previously described (20). The Lyn11-mNeonGreen IRES NLuc-arrestin-3 construct was described previously (54).

### Cell authentication, culture, and transfection

Cell authentication certificates are available on request. HEK293T and Flp-In T-REx 293 cells stably expressing receptors of interest were authenticated by Northgene (Case Number C-24809a and C-24809b respectively). These lines were cultured in DMEM supplemented with 10% Foetal Bovine Serum (FBS) and penicillin-streptomycin in a 5% CO_2_ 37 °C incubator. Cell lines were tested for *mycoplasma* contamination every 3 months and were confirmed to be *mycoplasma* free throughout the period of the studies. Transfections were performed using polyethylenimine 25,000 MW (PEI) at a 6:1 PEI:DNA ratio, scaled to a 10 cm dish equivalent. For TRUPATH-like transfections into HEK293T cells total DNA used was 6 μg, comprising 5 μg of the pIRES sensor construct and varying amounts of FLAG-hGPR35a plasmid DNA. Empty plasmid (pcDNA5.1/TO/FRT) was used to equalize the DNA amount across all samples. For arrestin bystander BRET experiments, Flp-In T-REx 293 cells were transfected with 5 μg of the Lyn11-mNeonGreen IRES NLuc-arrestin-3 plasmid DNA only.

Human colorectal adenocarcinoma HT29 cells (authentication by European Collection of Authentication Cell Cultures) (ECACC) were grown in McCoy's 5A (Modified) Medium (Gibco 16,600–082) supplemented with 10% FBS and 1 × penicillin/streptomycin. Human hepatocellular carcinoma HepG2 (authentication by American Type Culture Collection, ATCC) and HepG2 GPR35 KO cells (31) were grown in Minimum Essential Medium (MEM) supplemented with 10% FBS, 2 mM l-glutamine, 1 × non-essential amino acid, 1 mM sodium pyruvate, and 1 × penicillin/streptomycin. Experiments were performed when cell confluence reached 80%.

### xCELLigence assay

xCELLigence Real-Time Cell Analysis (https://www.agilent.com/en/product/cell-analysis/real-time-cell-analysis) was used to monitor the activation state of GPR35. HT29 or HepG2 cells (either WT or GPR35 KO) cells were seeded onto xCELLigence E plates (Agilent, 300600910) (HT29 cells 6 × 10^4^ cells/well; HepG2 cells 2 × 10^4^ cells/well) and incubated for 24 h at 37 °C. To evaluate GPR35 activation or suppression, cells were exposed to lodoxamide, CID-2745687 or vehicle. Alterations in impedance (represented as ‘cell index’) was recorded every 15 s over a 1 h period.

### BRET assays

BRET experiments were conducted 48 h post-transfection. Cells were washed twice with HBSS buffer (Gibco, 14025092) supplemented 20 mM HEPES, pH 7.4, and incubated at 37 °C for 20 min. Coelenterazine-h (5 μM final concentration, NanoLight, 301) was added, and the plate was read using a PHERAStar plate reader. Baseline readings were taken 5 min after substrate addition, followed by ligand addition and further were taken readings 5 min post-ligand addition. In kinetic experiments, plates were read repeatedly over 30 min post ligand addition. BRET ratios were calculated from 530 nm channel/475 nm channel raw values.

### BRET assay calculations

For TRUPATH-style experiments, the BRET ratio from NLuc alone (without any co-expressed acceptor) was subtracted to determine the BRET signal. For ligand-independent TRUPATH-style experiments, the BRET signal was normalized to ‘no receptor DNA’ for HEK293T cells or ‘no DOX’ for Flp-In T-REx 293 cells. For ligand-mediated TRUPATH-style experiments, the BRET signal was normalized to vehicle response. For arrestin bystander BRET experiments BRET ratios were normalized to the maximum lodoxamide response. Statistical tests were performed using unpaired and two-tailed Student’s *t* test.

### Isolation of colonic crypts for organoids

Conditioned media (CM100) for organoid culture was made from L-WRN cells (ATCC, CRL-3276) as described by Miyoshi and Stappenbeck (2013). Briefly, confluent L-WRN cells were given fresh ADF + daily for 5 days. Each day the “spent” medium was collected, pooled, and centrifuged to remove cells. CM50 was prepared by mixing equal parts CM100 and ADF + supplemented with 50 μM EGF (ThermoFisher, PMG8041) and primocin (Invivogen, ant-pm-1).

Colonic crypts were isolated following a modified protocol of Fan *et al.*, (55). Colons from wild-type, GPR35 knock out and hGPR35a-HA transgenic mice were cleaned, everted, and incubated in PBS containing 20 mM EDTA at 37 °C for 30 min. Crypts were dislodged by shaking and spun down at 100g for 2 min. Crypts were resuspended in CM100 medium (with 100 μM EGF, 20 μM Y-27632) (HelloBio, HB2297) and mixed with an equal volume of Matrigel (Corning, 356,237). Matrigel domes containing approximately 500 crypts per 25 μl were seeded in 24-well plates and incubated upside-down for 15 min to allow the Matrigel to set before adding CM50 medium (with 10 μM Y-27632).

### Organoid permeability assays

Organoid permeability assays were adapted from Bardenbacher, *et al.* (56). Briefly, organoids were split into near-single-cell clumps and embedded in Matrigel for 1 to 2 days. The organoids were then gently removed from Matrigel, re-embedded in fresh Matrigel domes in 8-well chamber slides and allowed to settle on ice for 5 min before incubation. One day before the experiment, ligands (lodoxamide 1 μM or CID-2745687, 10 μM) or vehicle (DMSO, final 0.1% (v/v)) were added. For FITC-dextran experiments, FITC-dextran (0.1 mg/ml final concentration) was added immediately after ligand addition. For sodium fuorescein (NaFluo) experiments NaFluo (0.05 mg/ml final concentration) was added 70 min before imaging. Organoids were imaged using a Zeiss LSM 880 confocal microscope with a 20X objective and a 488 nm laser. Fluorescence intensity was measured inside and outside each organoid (n = 20–30 per sample), and the relative internal concentration (Ratio = F_in_/F_out_) was calculated. Organoid diameter was measured, and the apparent permeability coefficient (Papp) was calculated as Papp = (Ratio × Vol)/SA, where Vol is organoid volume and SA is surface area. Each experiment was repeated at least 3 times, data presented is mean of the means of each experiment. Statistical tests were performed using either one-way ANOVA or Student’s *t* test unpair, two-tailed.

### Immunohistochemistry

Colons from wild-type and hGPR35a-HA transgenic mice were isolated, cleaned, and fixed in 4% paraformaldehyde overnight. The tissues were then transferred to 70% ethanol, paraffin-embedded, and sectioned. Colonoids, fixed for 20 min in 4% PFA, and sections were washed with PBS (pH 7.4, 0.1% Triton-X 100), blocked with 5% BSA and 10% goat serum overnight at 4 °C, and incubated with 1:100 rat anti-HA (Roche, 11867423001) overnight, or MUC2 (Novus, NBP1-31231, 1:1000) or ZO-1 (ProteinTech, 21773-1-AP, 1:2000) primary antibodies at RT for 1 h. After washing, sections and organoids were incubated with 1:200 goat anti-rat secondary antibody (Alexa Fluor 488, ThermoFisher, A-21434, or Alexa Fluor 594, AbCam ab150160) or goat anti-rabbit Alexa Fluor 488 secondary antibodies (AbCam, ab150077) for 2 h, washed again, and sections mounted with VECTASHIELD Antifade Mounting Medium with DAPI (Vectorlabs, H-1200–10). Organoids were stained with DAPI and washed before mounting whole in sucrose:glycerol mounting medium. Images were captured using a Zeiss LSM 880 confocal microscope or an EVOS fluorescence microscope.

### Western blotting

Flp-In T-REx 293 cells were grown to near confluence and treated with the indicated concentration of DOX overnight. Cells were washed with cold PBS and lysed using RIPA buffer (50 mM TRIS, 150 mM NaCl, 0.5% Na-Deoxycholate, 1% Igepal, 0.1% SDS) with a protease inhibitor cocktail (1 tablet per 10 ml, Roche, 5892970001). Lysates were passed through a 25G needle and protein concentrations measured using a BCA kit (ThermoFisher, 23,225). Lysates (20 μg) were mixed with Laemmli buffer (Sigma, 53,401-1VL) resolved on SDS-PAGE 4 to 12% gels (ThermoFisher, NP0321BOX), transferred to nitrocellulose membranes (ThermoFisher, 88,018), washed with 0.1% Tween-20 PBS, and blocked with 10% BSA in the same wash buffer for 2 h. Membranes were stained with 1:20,000 rat anti-FLAG (ProteinTech, 20543-1-AP) overnight, washed, and stained with 1:10,000 donkey anti-rat secondary antibody (LI-COR Biosciences, 926–32213) for 2 h. Membranes were imaged using a LiCor Odyssey imaging system.

### In-Cell Western

Cells were seeded into poly-D-lysine-coated clear-bottom black 96-well plates and incubated overnight. The next day, cells were treated with DOX for 24 h. Cells were fixed with 4% PFA, washed with PBS, and stored at 4 °C. On the day of staining, cells were washed with PBS containing 0.2% Tween-20 (In-Cell wash buffer) and permeabilized. Blocking was performed with 10% BSA in In-Cell wash buffer for several hours, followed by staining with 1:2000 rat anti-FLAG antibody overnight at 4 °C. After washing, cells were incubated with donkey anti-rat secondary antibody and Cell-Tag 700 (LI-COR Biosciences, 926–4109). Fluorescence intensity was measured using a LiCor Odyssey. Anti-FLAG measurements were normalized to Cell-Tag 700 values.

### Animal maintenance

Wild type and transgenic hGPR35a-HA knock-in (31) male C57Bl/6N mice were used in this study. Breeding, maintenance, and sacrifice of mice conformed to the United Kingdom Home Office regulations (PPL number: PP0894775).

## Data availability

The transgenic hGPR35a-HA knock-in mouse line is available upon request to either ABT or GM. All other data and reagents are freely available from GM (Graeme.Milligan@glasgow.ac.uk) or ABT (Andrew.Tobin@glasgow.ac.uk) or or through the University of Glasgow’s online data repository.

## Supporting information

This article contains supporting information.

## Conflict of interest

The authors declare no conflict of interest with the content of the article.

## Figures and Tables

**Supplemental Figure 4:**