# Supplementary material for: Biased constitutive signaling of the G protein-coupled receptor GPR35 suppresses gut barrier permeability[image]
Source: J Biol Chem. 2024 Nov 29;301(1):108035. doi: 10.1016/j.jbc.2024.108035 (PMC11732441; doi:10.1016/j.jbc.2024.108035)
Supplement: Supplemental Figure Legends [file mmc1.docx]

**Supplemental Figure Legends**

**Biased constitutive signalling of the G protein-coupled receptor GPR35 suppresses**

**gut barrier permeability**

**Tezz Quon^1^, Li-Chiung Lin^1^, Amlan Ganguly^1^, Brian D. Hudson^1^, Andrew B. Tobin^1^ and Graeme Milligan^1^**

^1^ Centre for Translational Pharmacology, School of Molecular Biosciences, College of Medical, Veterinary and Life Sciences, University of Glasgow, Glasgow G12 8QQ, Scotland, United Kingdom

**Address correspondence to Graeme Milligan, Centre for Translational Pharmacology, University of Glasgow, Glasgow G12 8QQ, Scotland, U.K. (+44 7738 121146) (**[**Graeme.Milligan@glasgow.ac.uk**](mailto:Graeme.Milligan@glasgow.ac.uk)**)**

GM and ABT designed and co-ordinated the programme of work and developed the mouse model, TQ, L-CL, and AG performed and analysed experiments and assisted with manuscript production. BDH provided advise on the use of sensor constructs. GM and ABT wrote initial drafts of the manuscript and all other authors contributed to the final text.

**Competing Interests** The authors declare no conflict of interests.

**Supplemental Figure 1.** **Expression of hGPR35a-HA and gut markers** **in colon and organoids**

(**A**) Formalin fixed, paraffin-embedded sections of colons from hGPR35a-HA knock-in and wild type mice, were stained with anti-HA antibody (red), to detect the receptor, and DAPI (blue) to define cell nuclei. These were then imaged on an EVOS microscope, scale bar = 50μm. Colon organoids in culture (**B**) generated from hGPR35a-HA knock-in mice maintained expression of MUC2 and ZO-1 (green) as detected by appropriate antisera. Samples were co-stained with DAPI (blue) and imaged using Zeiss 880 confocal microscope. Scale bars = 10μm.

**Supplemental Figure 2.** **hGPR35b promotes constitutive activation of both Gα_13_ and Gα_12_ but not Gα_o_ isoforms**

HEK293 cells were transiently transfected with the Gα_12_-containing heterotrimeric sensor (5μg) and the indicated amounts of FLAG-hGPR35a. BRET, reflecting the extent of association of the G protein heterotrimer was then measured (**A**). (**B**) HEK293 cells co-transfected with 31.6 ng FLAG-hGPR35a plasmid and the Gα_12_-containing sensor were exposed to vehicle (**open circles**), the GPR35 agonist lodoxamide (1 x 10^-6^M) (**dark circles**) or the hGPR35 inverse agonist CID-2745687 (1 x 10^-5^M) (**light circles**) and alteration in BRET signal (relative to vehicle treated) measured over time following addition of a luciferase substrate. (**C**) Varying amounts of the FLAG-hGPR35a plasmid were transfected into HEK293 cells along with a constant amount of the Gα_12_-containing TRUPATH sensor plasmid. Effects of CID-2745687 (**light circles**) or the hGPR35 agonist lodoxamide (**dark circles**) (each at 1 x 10^-5^M) on BRET signal were measured after 5 mins and compared to treatment with vehicle. Data are means +/- SD, n = 3.

**Supplemental Figure 3. Varying hGPR35b levels highlight receptor-induced constitutive activation of both Gα_13_ and Gα_12_ but not Gα_oA_**

A Flp-In T-REx 293 cell line stably harboring FLAG-hGPR35b was established. Following treatment of cells for 16 h with varying concentrations of doxycycline, expression of the receptor was assessed via In-Cell Western blots to detect the N-terminal (and hence extracellular) FLAG-tag cell (**A**). Following transient expression of Gα_13_/Gβ3/Gγ9 (**dark squares**), Gα_12_/Gβ3/Gγ9 (**dark circles**), or Gα_oA_/Gβ3/Gγ9 (**light circles**) into cells treated with the indicated concentrations of doxycycline (DOX), BRET was measured 10 mins after addition of the NLuc substate (**B**). Ligand-independent constitutive activity was observed for Gα_12_ and Gα_13_, but not for Gα_oA_ Data are means +/- SD, n = 3 and presented as NLuc corrected BRET units normalised to no DOX-induced FLAG-hGPR35b expression.

**Supplemental Figure 4. hGPR35b is only able to weakly activate Gα_oA_**

Lodoxamide (1 x 10^-6^M) promotes activation and dissociation of both (**A**) Gα_13_/Gβ3/Gγ9 (**dark circles**) and (**B**) Gα_12_/Gβ3/Gγ9 (**dark squares**) in Flp-In T-REx 293 cells able to inducible express hGPR35b. In comparison activation of (**C**) Gα_oA_/Gβ3/Gγ9 (**light circles**) was very limited. NLuc corrected BRET units normalized as % change from vehicle. Data are mean +/ - SD, n = 4.
